# Supplementary material for: On the possibility of carbon-free heteropolymers on Venus: a computational astrobiology study
Source: QRB Discov. 2025 Sep 25;6:e23. doi: 10.1017/qrd.2025.10012 (PMC12554815; doi:10.1017/qrd.2025.10012)
Supplement: Madan et al. supplementary material [file S2633289225100124sup001.pdf]

Supplementary Information for

On the Possibility of Carbon-free Heteropolymers on Venus: A Computational Astrobiology Study

Ishaan Madan, Shekoufeh A. Aliabadi, Johanna Huhtasaari, Ebba Matic, Emil Hogedal, Kinga Kamińska, Filip Nilsson, Axel Stark, Fernando Izquierdo Ruiz, Hilda Sandström, Martin Rahm*

All raw computational output files with necessary numerical data to reproduce any of the calculations in this work are openly available on Zenodo at https://doi.org/10.5281/zenodo.15881845.

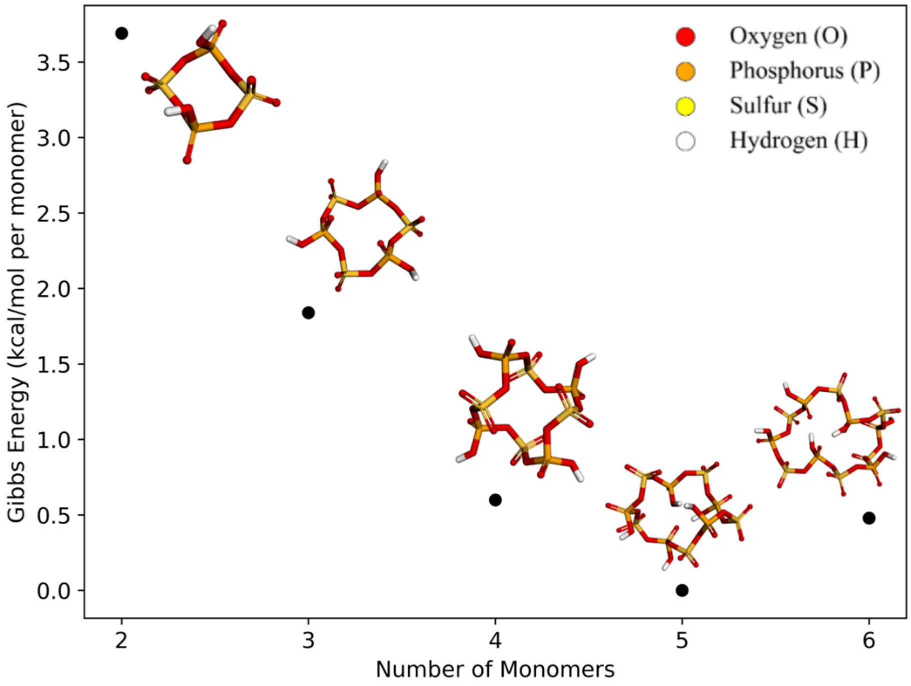

Figure S1. Gibbs free energy per monomer of screened cyclic polymers relative to the lowest-energy polymer. The lowest energy structures, the pentamer and hexamer, are omitted from thermodynamic consideration in the main text, as their energies are artificially lowered due to intramolecular H-bonding. The H atoms are here only models intended to emulate larger R groups of the envisioned polymer.

Table S1. Bond dissociation enthalpy, ΔH_r, calculated for model monomer **2**. Enthalpies for homolytic and heterolytic cleavage are reported for vacuum and sulfuric acid environments.

Solvation	Cleavage Type	Reaction	Bond Type	BDE (kcal/mol)
Vacuum	Homolytic Cleavage	**2** → $HSO_3 + H_2PO_4$	S–O	63
		**2** → $HSO_4 + H_2PO_3$	P–O	94
	Heterolytic Cleavage	**2** → $HSO_3^+ + H_2PO_4^-$	S–O	199
		**2** → $HSO_3^- + H_2PO_4^+$		236
		**2** → $HSO_4^+ + H_2PO_3^-$	P–O	312
		**2** → $HSO_4^- + H_2PO_3^+$		181
Solvated	Homolytic Cleavage	**2** → $HSO_3 + H_2PO_4$	S–O	60
		**2** → $HSO_4 + H_2PO_3$	P–O	91
	Heterolytic Cleavage	**2** → $HSO_3^+ + H_2PO_4^-$	S–O	73
		**2** → $HSO_3^- + H_2PO_4^+$		110
		**2** → $HSO_4^+ + H_2PO_3^-$	P–O	191
		**2** → $HSO_4^- + H_2PO_3^+$		51

Table S2. Calculated Gibbs free reaction energies. Values are corrected for concentrations, solvation effects and temperatures relevant for Venusian cloud-like conditions, as explained in the Methods section.

Reaction Type	Reaction	$\Delta G^\circ_{corrected}$ (kcal/mol)
Dehydration synthesis	$\mathbf{2} + (SO_3POOR)_1 \rightarrow (SO_3POOR)_2 + H_2O$	11
	$\mathbf{2} + (SO_3POOR)_2 \rightarrow (SO_3POOR)_3 + H_2O$	-1
	$\mathbf{2} + (SO_3POOR)_3 \rightarrow \mathbf{1} + H_2O$	-2
	$\mathbf{2} + \mathbf{1} \rightarrow (SO_3POOR)_5 + H_2O$	-1
Formation of monomer (**2**)	$H_3PO_4 + H_2SO_4 \rightarrow \mathbf{2} + H_2O$	1
	$H_3PO_4 + SO_3 \rightarrow \mathbf{2}$	-3
	$HPO_3 + H_2SO_4 \rightarrow \mathbf{2}$	-23
Formation of polymer (**1**)	$4\,HPO_3 + 4\,H_2SO_4 \rightarrow \mathbf{1} + 4\,H_2O$	-84
	$4\,HPO_3 + 4\,SO_3 \rightarrow \mathbf{1}$	-101
	$4\,H_3PO_4 + 4\,SO_3 \rightarrow \mathbf{1} + 4\,H_2O$	-3
Water Removal	$H_2O + SO_3 \rightarrow H_2SO_4$	-4
	$2\,H_2O + SO_3 \rightarrow HSO_4^- + H_3O^+$	4
Hydrolysis	$\mathbf{1} + 8\,H_2O \rightarrow 4\,H_2SO_4 + 4\,H_3PO_4$	-13
Thermal Decomposition	$\mathbf{1} \rightarrow P_4O_{10} + 4\,SO_3 + 2\,H_2O$	13

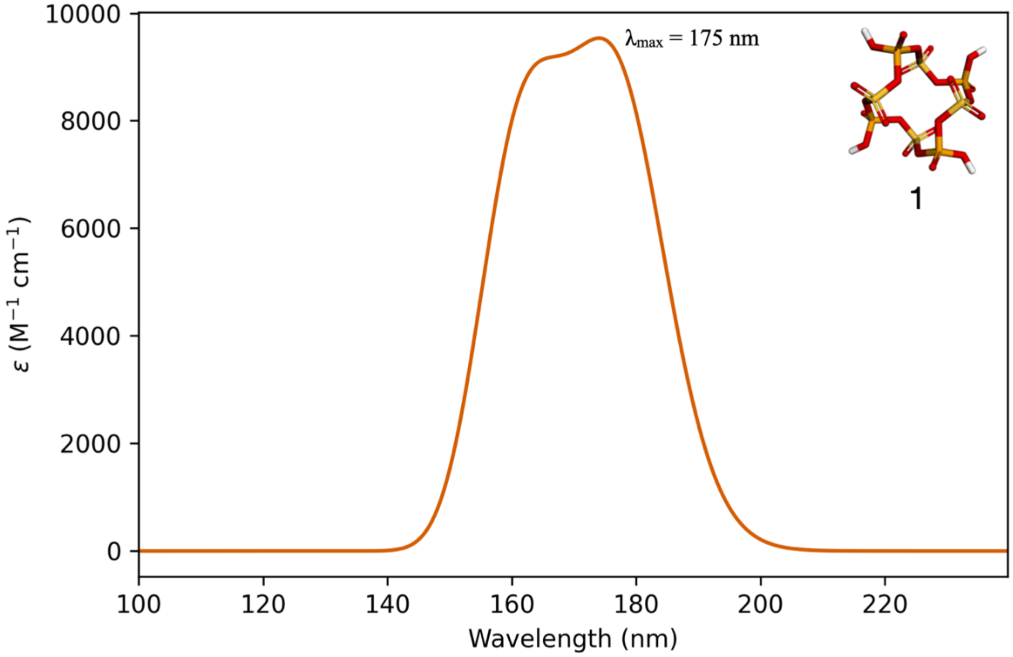

Figure S2. Simulated TD-DFT spectra for structure **1** (solvated). Strongest absorption occurs at 175 nm. The absence of allowed transitions at higher energies suggests that such polymers are unlikely to contribute to Venus's unknown near-UV absorber.

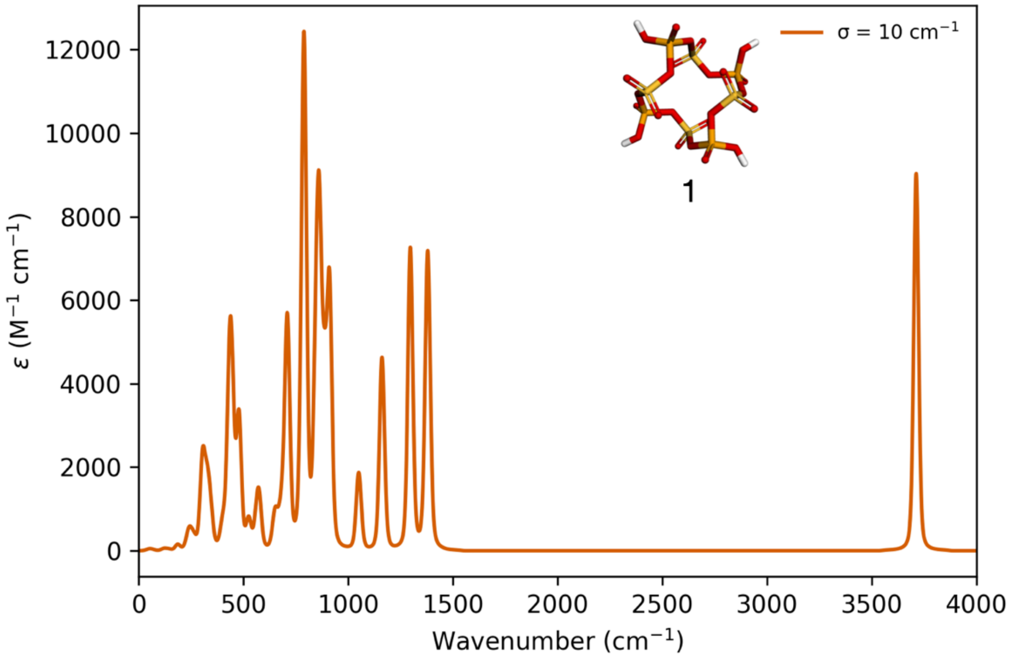

Figure S3. Simulated IR spectra of Structure **1** (solvated). Several diagnostic vibrational modes are identified (Table 2), notably unique P–O–S stretches absent in common reference compounds.

Table S3. Summary of relevant instruments, detection modalities, and suitability of planned Venus missions for identifying non-volatile, non-aromatic biopolymer analogs.

Mission	Instrument(s)	Detection Modality	P–O–S Detection Suitability
**EnVision** (Helbert et al., 2024)	VenSpec-H (1-2.5 µm high-res near-IR), VenSpec-M (0.3-1.2 multi-channel emissivity), VenSpec-U (UV imaging <0.2 µm)	IR, thermal, UV remote sensing	Not suitable. No coverage of mid-IR vibrational bands (P=O, S=O, P–O–S); global mapping only
**DAVINCI** (Garvin et al., 2022)	VMS (QMS, 2-550 Da unit-mass resolution, EI ionization), VTLS (3-channel tunable laser spectrometer)	Mass spectrometry, laser spectroscopy	Not suitable. Unit-mass resolution and EI bias limit polymer identification; VTLS targets known gases only
**Morning Star** (French et al., 2022)	Autofluorescence nephelometer (440 nm laser; aerosol size/shape + fluorescence)	Laser-induced fluorescence scattering	Not suitable
**VERITAS** (Smrekar et al., 2022)	VEM (1.02, 1.10, 1.18 µm night-side near-IR mapper), SAR radar, gravity science	NIR emissivity, radar, radio-science	Not suitable. Remote sensing only; no in situ spectroscopy or mass spec for cloud particles
**Venera-D** (Zasova et al., 2019)	Orbiter IR + UV spectrometers; Lander GC-MS (volatiles ≤ 200 Da), XRF (bulk elemental analysis)	Remote sensing; in situ MS/XRF	Not suitable. Lander targets surface; GC-MS optimized for small volatiles, not large polymers
**Shukrayaan-1** (Sundararajan, 2021)	Imaging spectrometer (UV-IR windows), radio science experiment	Remote UV/IR spectroscopy, radio occultation	Not suitable. No dedicated cloud-particle instrument; no mass spec or mid-IR detection

SI References

French, R., Mandy, C., Hunter, R., Mosleh, E., Sinclair, D., Beck, P., Seager, S., Petkowski, J. J., Carr, C. E., Grinspoon, D. H., Baumgardner, D., & on behalf of the Rocket Lab Venus Team. (2022). Rocket Lab mission to Venus. *Aerospace*, *9*(8), 445. https://doi.org/10.3390/aerospace9080445

Garvin, J. B., Getty, S. A., Arney, G. N., Johnson, N. M., Kohler, E., Schwer, K. O., Sekerak, M., Bartels, A., Saylor, R. S., Elliott, V. E., Goodloe, C. S., Garrison, M. B., Cottini, V., Izenberg, N., Lorenz, R., Malespin, C. A., Ravine, M., Webster, C. R., Atkinson, D. H., … Zolotov, M. (2022). Revealing the mysteries of Venus: The DAVINCI mission. *The Planetary Science Journal*, *3*(5), 117. https://doi.org/10.3847/psj/ac63c2

Helbert, J., Vandaele, A.-C., Marcq, E., Tackley, P., Machado, P., Min, M., Ferus, M., Vinatier, S., Lasue, J., Lara, L. M., Widemann, T., Robert, S., Alemanno, G., Erwin, J., Neefs, E., Bertran, S., Lustrement, B., Hagelschuer, T., Peter, G., … VenSpec Team. (2024). *The VenSpec suite on the ESA Envision mission – a holistic investigation of the coupled surface atmosphere system of Venus*. https://doi.org/10.5194/epsc2024-423

Smrekar, S., Hensley, S., Nybakken, R., Wallace, M. S., Perkovic-Martin, D., You, T.-H., Nunes, D., Brophy, J., Ely, T., Burt, E., Dyar, M. D., Helbert, J., Miller, B., Hartley, J., Kallemeyn, P., Whitten, J., Iess, L., Mastrogiuseppe, M., Younis, M., … Mazarico, E. (2022, March 5). VERITAS (Venus emissivity, radio science, InSAR, topography, and spectroscopy): A discovery mission. *2022 IEEE Aerospace Conference (AERO)*. 2022 IEEE Aerospace Conference (AERO), Big Sky, MT, USA. https://doi.org/10.1109/aero53065.2022.9843269

Sundararajan, V. (2021, November 15). Tradespace exploration of space system architecture and design for India's shukrayaan-1, Venus orbiter mission. *ASCEND 2021*. ASCEND 2021, Las Vegas, Nevada & Virtual. https://doi.org/10.2514/6.2021-4103

Zasova, L. V., Gorinov, D. A., Eismont, N. A., Kovalenko, I. D., Abbakumov, A. S., & Bober, S. A. (2019). Venera-D: A design of an automatic space station for Venus exploration. *Solar System Research*, *53*(7), 506–510. https://doi.org/10.1134/s0038094619070244
